# Effects of selenium enrichment on fermentation characteristics, selenium content and microbial community of alfalfa silage

**DOI:** 10.1186/s12870-024-05268-1

**Published:** 2024-06-14

**Authors:** Pengbo Sun, Gentu Ge, Lin Sun, Shuai Du, Yichao Liu, Xingquan Yan, Jiawei Zhang, Yuhan Zhang, Zhijun Wang, Yushan Jia

**Affiliations:** 1 Key Laboratory of Forage Cultivation, Processing and High Efficient Utilization, Ministry of Agriculture, Beijing, People’s Republic of China; 2 Key Laboratory of Grassland Resources, Ministry of Education, Beijing, People’s Republic of China; 3 College of Grassland, Resources and Environment, Inner Mongolia Agricultural University, Hohhot, China; 4 Inner Mongolia Academy of Agricultural and Animal Husbandry Sciences, Hohhot, China; 5 Ordos Institute of Forestry and Grassland Science, Ordos, China; 6 Forestry and Grassland Work Station of Inner Mongolia, Hohhot, China

**Keywords:** Alfalfa, Selenium, Biofermentation, Selenium speciation, In vitro digestion

## Abstract

**Background:**

Selenium is essential for livestock and human health. The traditional way of adding selenium to livestock diets has limitations, and there is a growing trend to provide livestock with a safe and efficient source of selenium through selenium-enriched pasture. Therefore, this study was conducted to investigate the effects of selenium enrichment on fermentation characteristics, selenium content, selenium morphology, microbial community and in vitro digestion of silage alfalfa by using unenriched (CK) and selenium-enriched (Se) alfalfa as raw material for silage.

**Results:**

In this study, selenium enrichment significantly increased crude protein, soluble carbohydrate, total selenium, and organic selenium contents of alfalfa silage fresh and post-silage samples, and it significantly decreased neutral detergent fiber and acid detergent fiber contents (*p* < 0.05). Selenium enrichment altered the form of selenium in plants, mainly in the form of SeMet and SeMeCys, which were significantly higher than that of CK (*p* < 0.05). Selenium enrichment could significantly increase the lactic acid content, reduce the pH value, change the diversity of bacterial community, promote the growth of beneficial bacteria such as *Lactiplantibacillus* and inhibit the growth of harmful bacteria such as *Pantoea*, so as to improve the fermentation quality of silage. The in vitro digestibility of dry matter (IVDMD), in vitro digestibility of acid detergent fibers (IVADFD) and in vitro digestibility of acid detergent fibers (IVNDFD) of silage after selenium enrichment were significantly higher than those of CK (*p* < 0.05).

**Conclusion:**

This study showed that the presence of selenium could regulate the structure of the alfalfa silage bacterial community and improve alfalfa silage fermentation quality. Selenium enrichment measures can change the morphology of selenium in alfalfa silage products, thus promoting the conversion of organic selenium.

## Introduction

The world is facing the problem of “hidden hunger” caused by mineral deficiencies in food and the resulting health risks [1, 2]. Selenium, as an essential micronutrient for animals and humans, is important for the maintenance of normal life activities [3]. Selenium is a component of a number of important antioxidant enzymes that can help resist cellular damage due to free radicals, protect cells from oxidative stress, enhance the immune response, and improve the body’s resistance to pathogens and infections [4, 5]. Therefore, moderate amounts of selenium can promote livestock performance, enhance reproduction and resistance to disease. However, worldwide forages often contain insufficient levels of essential minerals to meet the needs of farmed livestock, resulting in low levels of organic selenium in animals [6]. To address this problem, selenium has been added to diets in an attempt to increase organic selenium levels in livestock [7, 8]. But, the range between the selenium nutritional dose and the maximum safe intake is very narrow, therefore excessive intake of selenium can easily cause toxic reactions [9, 10]. In general, livestock supplementation with selenium at levels between 0.30 and 1.00 mg.kg^− 1^ DM is described as adequate, and 3.00–4.00 mg.kg^− 1^ DM is described as high [6]. However, different species of selenium have different toxicity thresholds for livestock. In the past, in order to supplement selenium to livestock, most of them added selenate or selenite to the diet, but this traditional way of selenium supplementation always faces problems of poor safety and low absorption efficiency [11]. It is gradually becoming a trend to produce selenium-enriched animal products to provide selenium for human beings through the plant’s own selenium-enriched ability to convert exogenous selenium into organic selenium to provide safe and effective selenium to livestock.

Alfalfa (*Medicago sativa* L.) is widely used to feed livestock as a forage with high protein, strong selenium-rich ability and comprehensive nutritional value [12–14]. Alfalfa is often processed into two commodities, namely hay and silage. Alfalfa hay loses nutrients and nutritional value during production, transportation and storage due to various external factors [15]. Moreover, alfalfa hay is easily affected by the weather during processing, and it is easy to have mold and other problems when it encounters rain and other unfavorable environments [16]. Processing alfalfa into silage can avoid the above problems, and silage can better preserve protein and improve the palatability of livestock [17]. However, alfalfa is not easy to be silaged successfully due to its low carbohydrate and high buffer energy value [18, 19]. Therefore, additives are often added to ensure the success of alfalfa silage. The benefits of Lactic Acid Bacteria (LAB) in silage have now been demonstrated in a large number of studies [20]. In the silage of fodder crops, it is often the lactic acid bacteria that dominate the whole fermentation process [21, 22]. It means that lactic acid bacteria need to multiply rapidly to produce lactic acid in the pre-fermentation period, so that the silage pH can be quickly reduced to the ideal state to minimize silage loss and ensure that the silage reaches a high quality level [20]. It has been found that the addition of LABs can accelerate the pre-silage fermentation process by inhibiting harmful microbial populations, thus ensuring a higher nutritional value of the silage [23]. Some specific LAB can produce ferulic acid esterase during the fermentation process, which can degrade the neutral detergent fiber of plants during the silage process, thus improving the quality of silage [24]. Research on forage silage in the traditional sense has mainly focused on how to ensure the preservation of forage proteins, dry matter and the degradation of fiber. However, the provision of essential micronutrients to livestock through silage as an effective delivery mechanism to improve their product quality has not been considered.

Since 1995, Calomme et al. found that various Lactobacillus species could convert selenium into selenocysteine and selenomethionine [25]. Many scholars began to work on screening microorganisms capable of converting selenium. Michael et al. [26] screened three kinds of lactic acid bacteria through medium that could convert sodium selenite into selenium available to domestic animals. Fernando et al. [27] studied that 96 strains of lactic acid bacteria (*Lactococcus*, *Lactobacillus*, *Enterococcus*, and *Fructobacillus*) could convert sodium selenite mainly to selenoamino acids, and eight of them had a conversion rate of more than 80% for sodium selenite. While Cristina et al. [28] found that the growth of *Lactobacillus reuteri* was limited in the presence of selenium. It is evident that selenium has both beneficial and detrimental effects on microorganisms [29]. In recent years, most of them have focused on how to screen strains that can convert selenite or selenate into organic selenium that can be utilized by livestock, while fewer studies have been reported on the silage of selenium-enriched forage. Therefore, in this study, exogenous selenium was sprayed on alfalfa at the growth stage, and the selenium-enriched alfalfa was subjected to silage to investigate the chemical composition, fermentation quality, selenium content, selenium morphology, in vitro digestibility, and microbial community changes, and to clarify the feasibility of selenium-enriched alfalfa silage.

## Methods

### Treatments and plant materials

The plant materials were the alfalfa variety ‘WL232HQ’. NPs-Se (mean size: 25 nm) was 5% NPs-Se stock solution, provided by Shenzhen Zhigao Military and Civilian Integration Equipment Technology Research Institute. The experiment was conducted at the test site in Harringer Town, Baotou City, Inner Mongolia, China (longitude 110°37″−110°27″ E − 40°05″−40°17″ N). The soil type is chestnut soil (Chinese soil taxonomy). The chemical properties of soil were: organic matter, 4.4 g.kg^− 1^; total nitrogen, 0.45 g.kg^− 1^; fast-acting phosphorus, 23.57 g.kg^− 1^; fast-acting potassium, 145.94 mg.kg^− 1^; pH, 7.4. Alfalfa seeded in May 2020.

The experiment was designed with two treatment groups, a selenium-enriched alfalfa treatment group by spraying a 50 mg.L^− 1^ nano-selenium solution three times during the growth stage of alfalfa (Se), and a control treatment group by spraying an equal amount of clear water (CK). The spray rate was 1000 L.hm^− 2^, the plot area was 20 m^2^ with three replications, and it was harvested in the initial flowering stage. (June 2023).

The mowed alfalfa was dried to about 65% moisture content, cut into about 2 cm with a cutting machine, used as silage raw material, added 1 × 10^6^ cfu.g^− 1^*Lactobacillus plantarum*, mixed well and then packed into polyethylene bags (about 300 g per bag) sealed with a small vacuum plastisol sealing machine, and carried out a small-package silage test, with three replicates set up for each treatment. All samples were stored at room temperature (temperature range, 20–25 ℃) and opened after 15, 30 and 60 d of natural fermentation to determine fermentation characteristics, chemical composition, selenium content and selenium morphology. Meanwhile, the bacterial community structure and in vitro fermentation parameters (Analog 48 h) were determined after 60 d of natural fermentation.

### Determination of chemical composition, fermentation characteristics and in vitro digestion

The alfalfa samples were dried at 65 °C for 48 h and the dry matter content was determined [30]. Crude protein (CP = total *N* × 6.25) content was determined by an automatic Kjeldahl nitrogen analyzer [31]. Acid detergent fiber (ADF) and neutral detergent fiber (NDF) content were determined by an ANKOM automatic fiber analyzer [32]. Water soluble carbohydrates (WSC) were determined by anthracene anthracene analyzer [33].

After the silage samples were opened, 10 g of the samples were weighed into a sterile polyethylene bag, 90 mL of sterile distilled water was added, and the filtrate was filtered using a sterile homogenizer and placed in a 50 mL centrifuge tube for the determination of pH, organic acids and ammoniacal nitrogen. pH was measured by a convenient pH meter (Model: LEICI pH S-3 C, Shanghai Yitian Scientific Instrument Co., Ltd., Shanghai, China). Lactic acid (LA) and acetic acid (AA) were determined using high performance liquid chromatography (Model: Waters e2695, Milford, MA, USA; column: Waters Symmetry C18; oven temperature, 50 ℃; mobile phase, 3 mmol.L^− 1^ perchlorate solution; flow rate, 1.0 ml.min^− 1^; flame photometric detector, 210 nm; sample size, 5.0 µl). Ammonia nitrogen (NH_3_-N) concentrations were determined by the phenol-hypochlorite method [34]. In vitro rumen fermentation was carried out using ANKOM DAISY II (Model: DAISY II, USA; Temperature range: standard setting constant temperature 39.5 ℃, temperature control accuracy ± 0.1 ℃; operating voltage 220 ~ 240 V, 50 ~ 60 Hz) in vitro simulated incubator following the method of Selçuk et al [35]. The formula for calculating in vitro digestibility:

In vitro digestibility of a nutrient (%) = [(weight of sample × content of a nutrient in the sample) - (weight of residue × content of a nutrient in the residue)]/ (weight of sample × content of a nutrient in the sample).

### Determination of selenium content and selenium speciation

Determination of total selenium content by hydride generation-atomic fluorescence spectrometry (HG-AFS) [36]. Determination of selenium speciation by high performance liquid chromatography-hydride generation atomic fluorescence spectrometry (HPLC-HG-AFS) [37]. Weigh 0.1–1.0 g (accurate to 0.001 g) of solid sample in a 15mL centrifuge tube, add 5 mL Tris-HCl, shake well, and then sonicate for 30 min. Add 50 mg of cellulase, then add 20 mg of proteinase K, shake well, and place horizontally in a gas-bath constant temperature oscillator, adjust the temperature to 50℃±2℃, rotate the speed of 250 r.min^− 1^, and incubate for 18 h. Finally, 20 mg of Protease XIV was added, incubated at 37℃±2℃ for 18 h, and centrifuged at 4℃ and 10000r.min^**− 1**^ for 30 min. The extract was passed through a 0.22 μm aqueous filtration membrane and left to be measured. High performance liquid chromatography (HPLC) conditions: Hamilton PRP-X100 (250 mm×4.1 mm×10 μm) column; mobile phase V (20 mmol.L^− 1^ aqueous hydrogen diamine phosphate, pH = 6.0): V (methanol) = 98:2; elution for 15 min; injection volume of 100 µL; the temperature of the column was room temperature. Hydride generation atomic fluorescence spectrometry instrument conditions: lamp current / auxiliary cathode: 100 mA / 45 mA; photomultiplier tube negative high voltage: 300 V; carrier gas flow rate: 300 mL.min^− 1^; shielding gas flow rate: 700 mL.min^− 1^; atomization temperature: 800 ℃; furnace height: 8 mm. Five standard selenoamino acids were used in this experiment and included the following: Se(IV) (selenite), Se(IV) (selenite), SeCys_2_ (selenocystine), MeSeCys (methylselenocysteine), and SeMet (selenomethionine) purchased from the National Institute of Metrology for Certified Reference Materials, Beijing, China.

### Sequencing and analysis of microbial diversity

Total microbial DNA was extracted and PCR amplified from alfalfa samples after 60 days of silage according to Liu et al [38]. Primers amplified in the highly variable region of V3-V4 were 338 F (5′-ACTCCTACGGGGAGGCAGCAG-3′) and 806 R (5′GGACTACHVGGGTWTCTAAT-3′). PCR products were recovered using 2% agarose gel, purified using Axy Prep DNA Gel Extraction Kit, Tris-HCl eluted and detected by 2% agarose electrophoresis. QuantiFluor™-ST was used for quantification, and the purified amplified fragments were used to construct PE 2*300 libraries according to the Illumina MiSeq platform standard operating procedures. Sequencing was performed using Illumina Miseq PE300 platform (Majorbio Bio-Pharm Technology Co., Ltd., Shanghai, China).

### Statistical analyses

Two-factor analysis of variance (ANOVA) for chemical composition, fermentation quality and selenium content of alfalfa using SAS 9.4 software (SAS Institute, Inc., Cary, NC, USA). Multiple range tests using Duncan’s were used to assess differences between treatments. Graphing with Origin version 2021 software. High-throughput sequencing data were performed using the online platform of the Majorbio I-Sanger Cloud Platform (www.i-sanger.com).

## Results

### The Chemical composition of fresh alfalfa

The chemical composition of the selenium-enriched alfalfa silage material is shown in Table 1. After treatment of alfalfa at the growth stage by nano-selenium, significant differences in dry matter (DM), acid detergent fiber (ADF), neutral detergent fiber (NDF), crude protein (CP), and water soluble carbohydrates (WSC) of silage material from alfalfa compared to control (*p* < 0.05). The DM, CP, WSC were significantly higher in Se than in CK (*p* < 0.05), which increased by 6.55%, 10.10%, and 16.77%, respectively. While ADF and NDF were significantly lower in Se than in CK (*p* < 0.05), which decreased by 2.99% and 4.53%, respectively.

**Table 1:** Effect of nan-selenium on the chemical composition of silage raw materials

Items	CK	Se	SEM	*p*-value
Dry matter (g.kg^− 1^)	300.93b	320.63a	4.74	0.007
Acid detergent fiber (g.kg^− 1^)	313.50a	304.13b	2.20	0.004
Neutral detergent fiber (g.kg^− 1^)	411.70a	393.07b	4.64	0.015
Crude protein (g.kg^− 1^)	223.50b	246.07a	5.17	0.001
Water soluble carbohydrate (g.kg^− 1^)	35.60b	41.57a	1.42	0.005

CK: not selenium-enriched; Se: selenium-enriched; SEM: standard error of mean. Identical lowercase letters in the same row indicate no significant difference between selenium treatments at the 0.05 level (*p* > 0.05)

### Fermentation characteristics and chemical composition of alfalfa silage

The dynamics of the chemical composition of alfalfa after silage by selenium enrichment are shown in Table 2. The interaction effect of days of ensiling and selenium treatment was significant for DM, CP and WSC (*p* < 0.05), but not for ADF and NDF (*p* > 0.05). Whereas, the main effects of selenium treatment and days of ensiling had significant effects on ADF and NDF (*p* < 0.05). As the number of days of ensiling increased, the chemical composition of alfalfa silage in all treatments showed a decreasing trend and reached the lowest value at 60 days, but the change rule of each chemical composition was inconsistent when the number of days of ensiling was from 15 days to 30 days. DM and ADF of CK had no significant effect at 15 and 30 days, and were significantly lower at 60 days than at 15 days. In contrast, the turning points of DM and ADF changes in Se preceded CK and were significantly lower at 30 days than at 15 days. The NDF, CP and WSC of CK and Se showed the same pattern of change with the increase of days of ensiling, and both of them were significantly lower than 15 days at 30 days. At 60 days, the ADF and NDF of Se were significantly lower than CK (*p* > 0.05), while the CP of Se was significantly higher than CK (*p* < 0.05). After 60 days of silage, the loss rates of DM, ADF, NDF, CP and WSC in the Se group were 17.90%, 12.55%, 7.73%, 15.69% and 45.95%, respectively. The loss rates of DM, ADF, NDF, CP and WSC in the CK group were 12.36%, 10.91%, 8.11%, 13.23% and 36.74%, respectively.

**Table 2:** Effect of selenium enrichment on the chemical composition of alfalfa silage

Items	Treatment	Ensiling Days			SEM	*p*-value		
		15	30	60		T	D	T*D
Dry matter (g.kg^− 1^)	CK	274.07Ab	268.7ABa	263.73Ba	1.70	0.1342	< 0.0001	0.0228
	Se	282.40Aa	267.27Ba	263.23Ba				
Acid detergent fiber (g.kg^− 1^)	CK	307.57Aa	293.33ABa	279.30Ba	3.53	< 0.0001	< 0.0001	0.2468
	Se	284.50Ab	272.37Bb	265.97Bb				
Neutral detergent fiber (g.kg^− 1^)	CK	397.57Aa	381.30Ba	378.33Ba	2.84	< 0.0001	< 0.0001	0.0890
	Se	374.83Ab	365.23Bb	362.70Bb				
Crude protein (g.kg^− 1^)	CK	208.63Ab	195.00Bb	193.93Bb	2.82	< 0.0001	< 0.0001	< 0.0001
	Se	229.13Aa	208.50Ba	207.47Ba				
Water soluble carbohydrate (g.kg^− 1^)	CK	24.98Ab	22.98Ba	22.52Ca	0.51	< 0.0001	< 0.0001	< 0.0001
	Se	28.26Aa	22.91Ba	22.47Ba				

CK: not selenium-enriched; Se: selenium-enriched; SEM: standard error of mean. Identical lowercase letters in the same column indicate no significant difference between selenium treatments at the 0.05 level (*p* > 0.05); Identical capital letters in the same row indicate no significant difference between ensiling days treatments at the 0.05 level (*p* > 0.05)

The dynamics of fermentation quality of alfalfa silage after selenium enrichment are shown in Table 3. The interaction effect of days of ensiling and selenium treatments was significant for pH, LA, and NH_3_-N (*p* < 0.05). The reciprocal effect of days of ensiling and selenium treatment did not significantly affect AA, whereas the main effect of days of ensiling had a significant effect on AA (*p* < 0.05). The pH of all treatments showed a decreasing trend with increasing days of ensiling and was significantly lower at 30 days than at 15 days, while it remained almost the same at 60 days. At the same number of days of ensiling, the pH of Se was higher than that of CK at 15 days of ensiling and significantly lower than that of CK after 30 days of ensiling. LA, AA, and NH_3_-N showed an increasing trend with the increase in the number of days of ensiling, and reached the maximum value at 60 days. Under the same number of days of ensiling, the LA of Se was significantly lower than that of CK at 15 days of ensiling, and significantly higher than that of CK at 30 and 60 days of ensiling (*p* < 0.05), with increased by of 3.16% and 3.93%, respectively. There was no significant difference in AA values between Se and CK. The NH_3_-N of Se was higher than that of CK at 15 days of ensiling, and the two were basically the same after 30 days of ensiling, with no significant difference.

**Table 3:** Effect of selenium enrichment on the fermentation characteristics of alfalfa silage

Items	Treatment	Ensiling Days			SEM	*p*-value		
		15	30	60		T	D	T*D
pH	CK	5.28Ab	4.95Ba	4.92Ba	0.06	< 0.0001	< 0.0001	< 0.0001
	Se	5.36Aa	4.79Bb	4.77Bb				
Lactic acid (g.kg^− 1^)	CK	38.63Ca	39.56Bb	41.00Ab	0.52	0.7456	< 0.0001	< 0.0001
	Se	35.93Cb	40.85Ba	42.61Aa				
Acetic acid (g.kg^− 1^)	CK	14.23Ca	17.27Ba	19.47Aa	0.53	0.7342	< 0.0001	0.7216
	Se	14.13Ca	17.44Ba	19.53Aa				
Ammonia-N (g.kg^− 1^)	CK	17.27Cb	18.37Ba	19.77Aa	0.22	0.0585	< 0.0001	0.0173
	Se	17.83Ca	18.57Ba	19.57Aa				

CK, not selenium-enriched; Se, selenium-enriched; SEM, standard error of mean. Identical lowercase letters in the same column indicate no significant difference between selenium treatments at the 0.05 level (*p* > 0.05); Identical capital letters in the same row indicate no significant difference between ensiling days treatments at the 0.05 level (*p* > 0.05)

### Dynamics of selenium content and selenium morphology

**Fig. 1:**
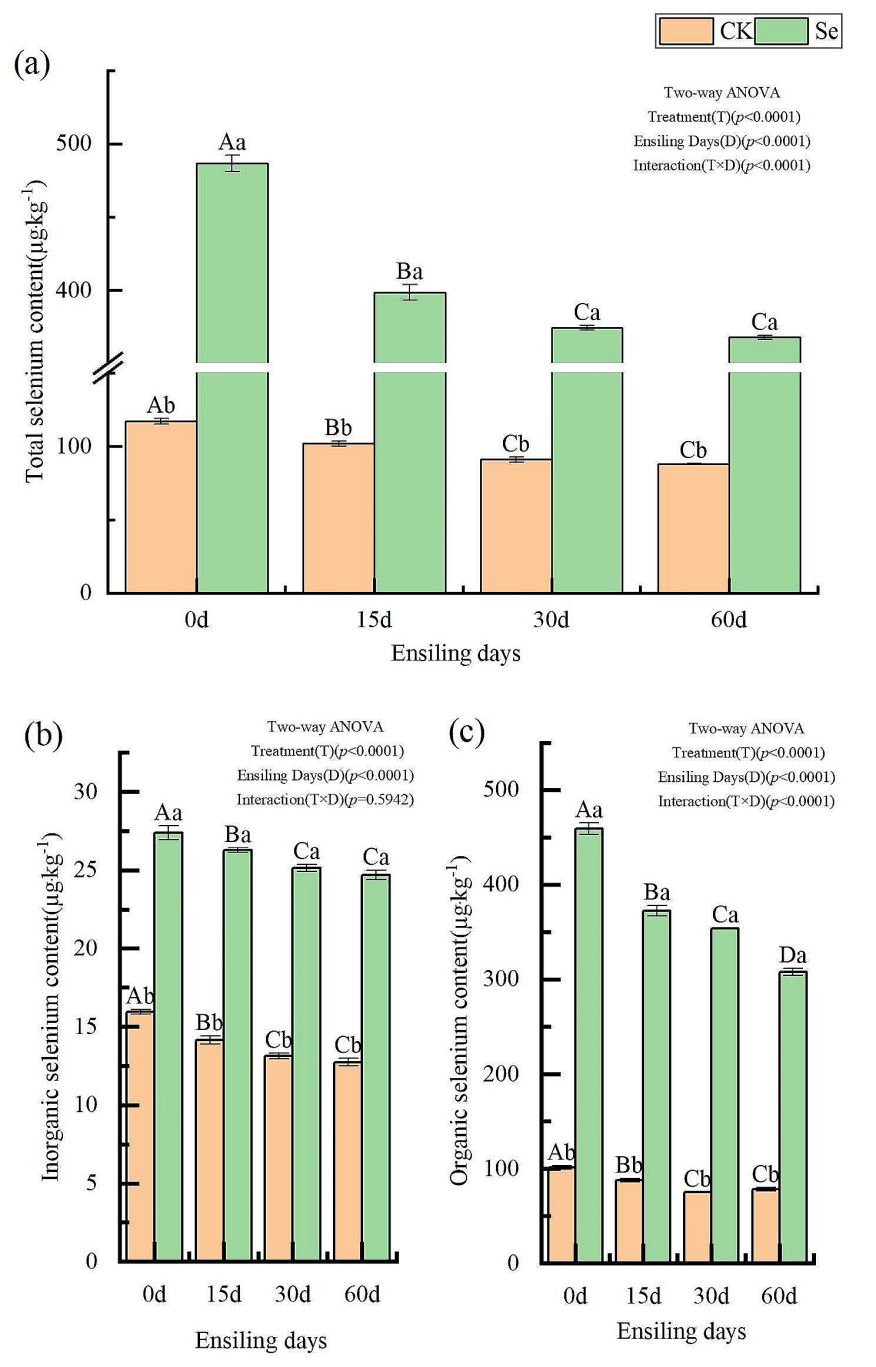
Effect of selenium enrichment on selenium content of alfalfa silage. CK, not selenium-enriched; Se, selenium-enriched; 0, raw materials for silage; 15, 15 days of ensiling; 30, 30 days of ensiling; 60, 60 days of ensiling. Identical lowercase letters indicate no significant difference between selenium treatments at the 0.05 level (*p* > 0.05); Identical capital letters indicate no significant difference between ensiling days treatments at the 0.05 level (*p* > 0.05)

The effects of selenium enrichment on the selenium content of alfalfa are shown in Fig. 1. The interaction effect of days of ensiling and selenium treatment was significant for total selenium and organic selenium (*p* < 0.05), but not for inorganic selenium (*p* > 0.05). But, the main effects of selenium treatment and ensiling days had significant effects on inorganic selenium content (*p* < 0.05). Selenium enrichment measures could significantly increase the total selenium, inorganic selenium and organic selenium contents of alfalfa, and the selenium content of the Se group was significantly higher than that of the CK group at all silage time periods (*p* < 0.05). At 60 days of silage, total selenium, inorganic selenium, and organic selenium contents of the Se group increased by 317.87%, 93.93%, and 294.13%, respectively, compared with the CK group. With the increase in the number of days of ensiling, the total selenium, and inorganic selenium contents of alfalfa in the two treatment groups changed in a consistent pattern, both showing a decreasing trend. The turning point for this change was 30 days of ensiling, and the changes in total and inorganic selenium content were not significant after 30 days of ensiling. At 60 days of silage, for total and inorganic selenium, the CK group decreased by 24.82% and 20.18%, respectively, and the Se group decreased by 24.35% and 9.81%, respectively. For organic selenium, the pattern of change in CK was consistent with the above description, but that of the Se group was still significantly lower at 60 days than at 30 days (*p* < 0.05). At this time, the CK and Se groups decreased by 22.78% and 32.92%, respectively.

**Fig. 2:**
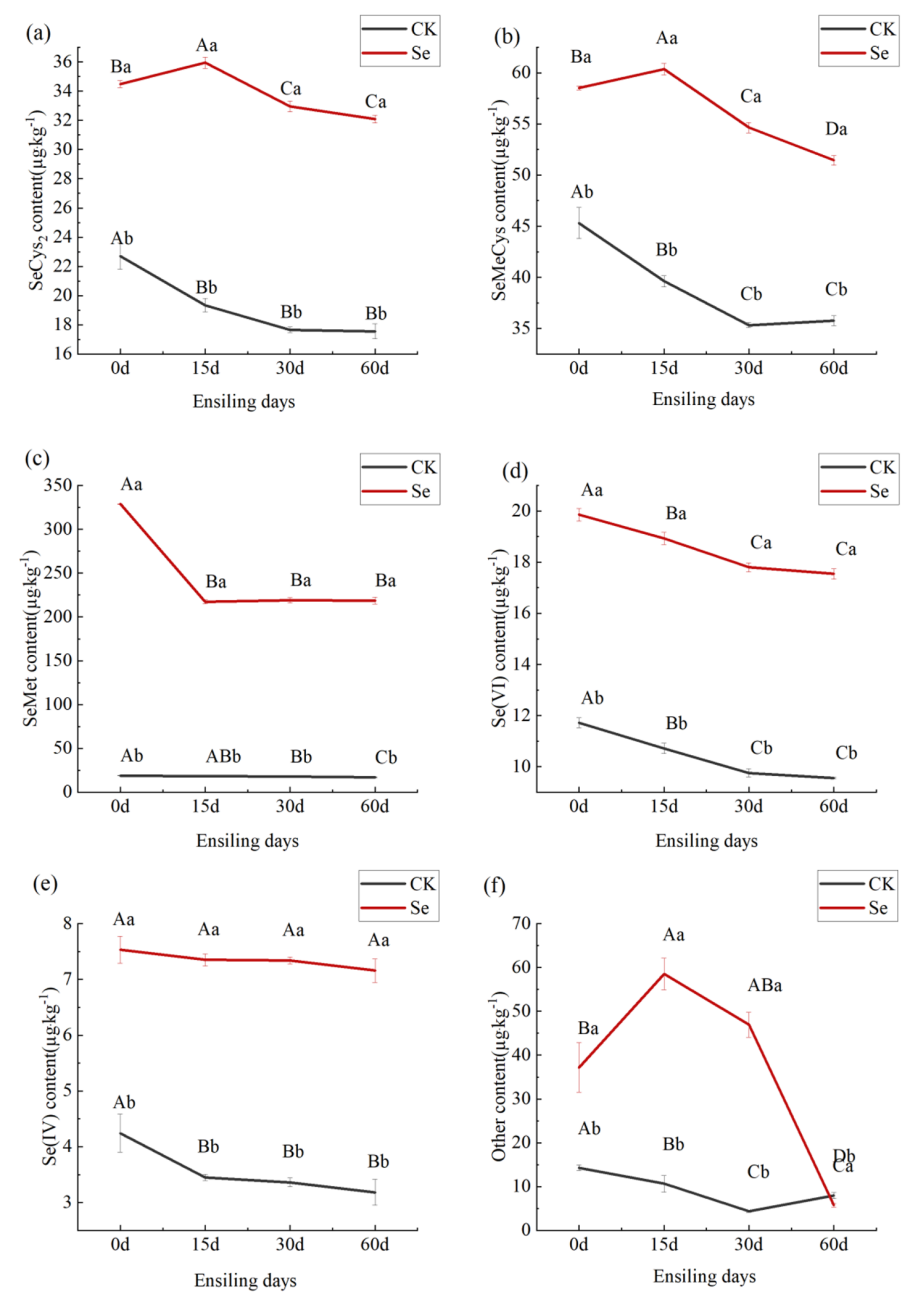
Effect of selenium enrichment on selenium morphology in alfalfa silage. (**a**), SeCys_2_, Selenocystine; (**b**), SeMeCys, Methylselenocysteine; (**c**), SeMet, Selenomethionine; (**d**) Se(VI), Hexavalent inorganic selenium; (**e**), Se(IV), Tetravalent inorganic selenium; (**f**), other, Other selenium forms. CK, not selenium-enriched; Se, selenium-enriched; 0, raw materials for silage; 15, 15 days of ensiling; 30, 30 days of ensiling; 60, 60 days of ensiling. Identical lowercase letters indicate no significant difference between selenium treatments at the 0.05 level (*p* > 0.05); Identical capital letters indicate no significant difference between ensiling days treatments at the 0.05 level (*p* > 0.05)

Changes in selenium morphology during selenium-enriched alfalfa silage are shown in Fig. 2. The contents of SeMeCys, SeCys_2_, SeMet, Se(IV), and Se(VI) were significantly higher in the Se group than in the CK group at all silage time periods (*p* < 0.05), and at 60 days of silage, increased by 43.89%, 82.78%, 1195.75%, 124.93% and 83.61%, respectively. Overall, there was a decreasing trend in the content of the different selenium forms with increasing silage time. The SeCys_2_, Se (IV), and Se(VI) contents of both treatment groups changed drastically before 15 days of ensiling, and were relatively stable after 30 days of ensiling, with a non-significant decreasing trend. For SeMeCys, the CK group showed a significant decreasing trend until 30 days of silage and stabilized at 60 days, while the Se group showed an increasing and then decreasing trend, with 15 days of silage being the turning point of this change, and a significant decreasing trend remained at 60 days of silage (*p* < 0.05). SeMet in the Se group decreased sharply before 15 days of ensiling and stabilized after 15 days. SeMet in the CK group had been decreasing slowly and was significantly lower at 60 days than at 30 days of ensiling (*p* < 0.05). The selenium content of other forms showed an opposite pattern of change in the two treatment groups, with a decreasing and then increasing trend in the CK group and an increasing and then decreasing trend in the Se group.

**Fig. 3:**
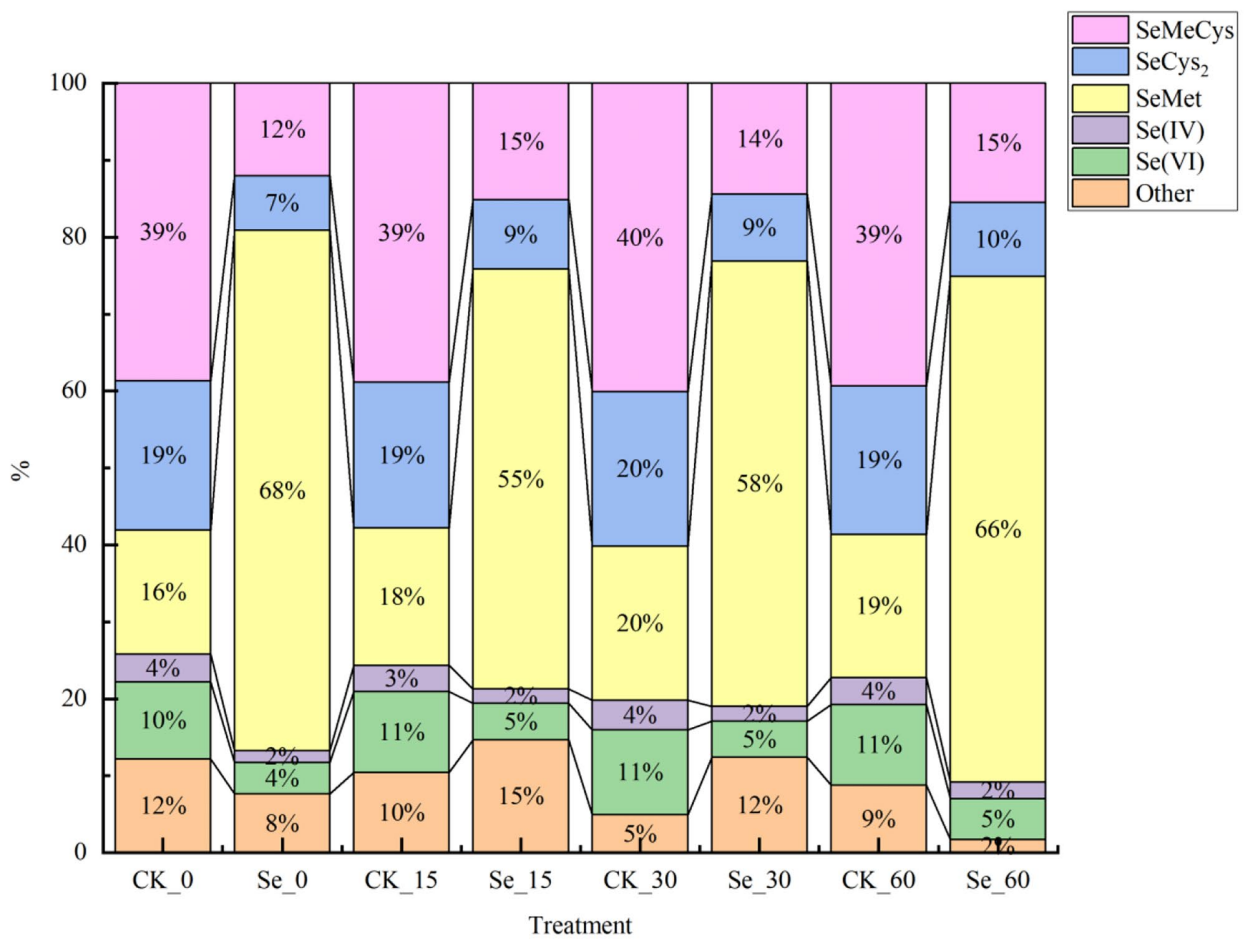
Effect of selenium enrichment on the percentage of selenium forms in alfalfa silage. CK, not selenium-enriched; Se, selenium-enriched; 0, raw materials for silage; 15, 15 days of ensiling; 30, 30 days of ensiling; 60, 60 days of ensiling

The proportion of various selenium forms in selenium-enriched alfalfa during silage is shown in Fig. 3. In the CK group, the selenium forms in alfalfa silage for 0 days were SeMeCys > SeCys_2_ > SeMet > other > Se (VI) > Se (IV) in the order of 39%, 19% 16%, 12%, 10% and 14%, respectively. In the Se group, SeMet > SeMeCys > other > SeCys_2_ > Se (VI) > Se(IV) were the selenium forms of alfalfa at 0 days of silage in the order of 68%, 12%, 8%, 7%,4% and 2%, respectively. With the increase of silage time, the percentage of SeMet in the CK group was rising, increasing by 3%, the percentage of other forms of selenium showed a decreasing trend, decreasing by 3%, and the percentage of SeMeCys, SeCys_2_, Se(IV), and Se(VI) did not change by more than 1%. In the Se group, the percentage of SeMeCys and SeCys_2_ increased by 3%, the percentage of SeMet decreased slightly, the percentage of Se (IV) and Se (VI) did not change significantly, and the proportion of other forms of selenium decreased significantly, from 8 to 2%, decreased by 6%.

### Bacterial diversity and community composition of alfalfa silage

**Fig. 4:**
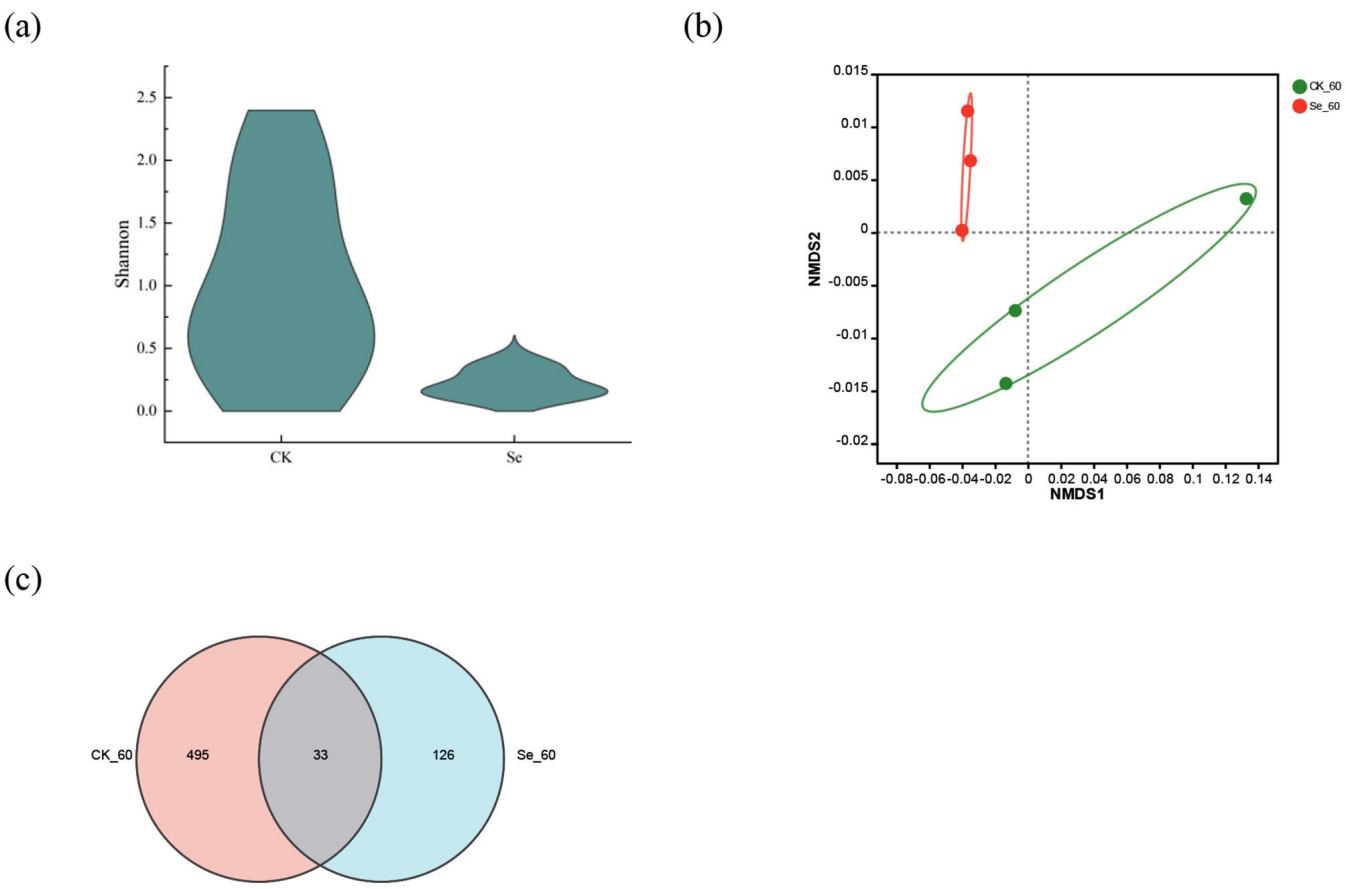
Analysis of bacterial diversity in alfalfa silage. (**a**), Shannon of bacterial alpha diversity; (**b**), Analysis of bacterial NMDS in alfalfa silage; (**c**), Bacterial OUT Venn in alfalfa silage. CK, not selenium-enriched; Se, selenium-enriched. 60, 60 days of ensiling

**Fig. 5:**
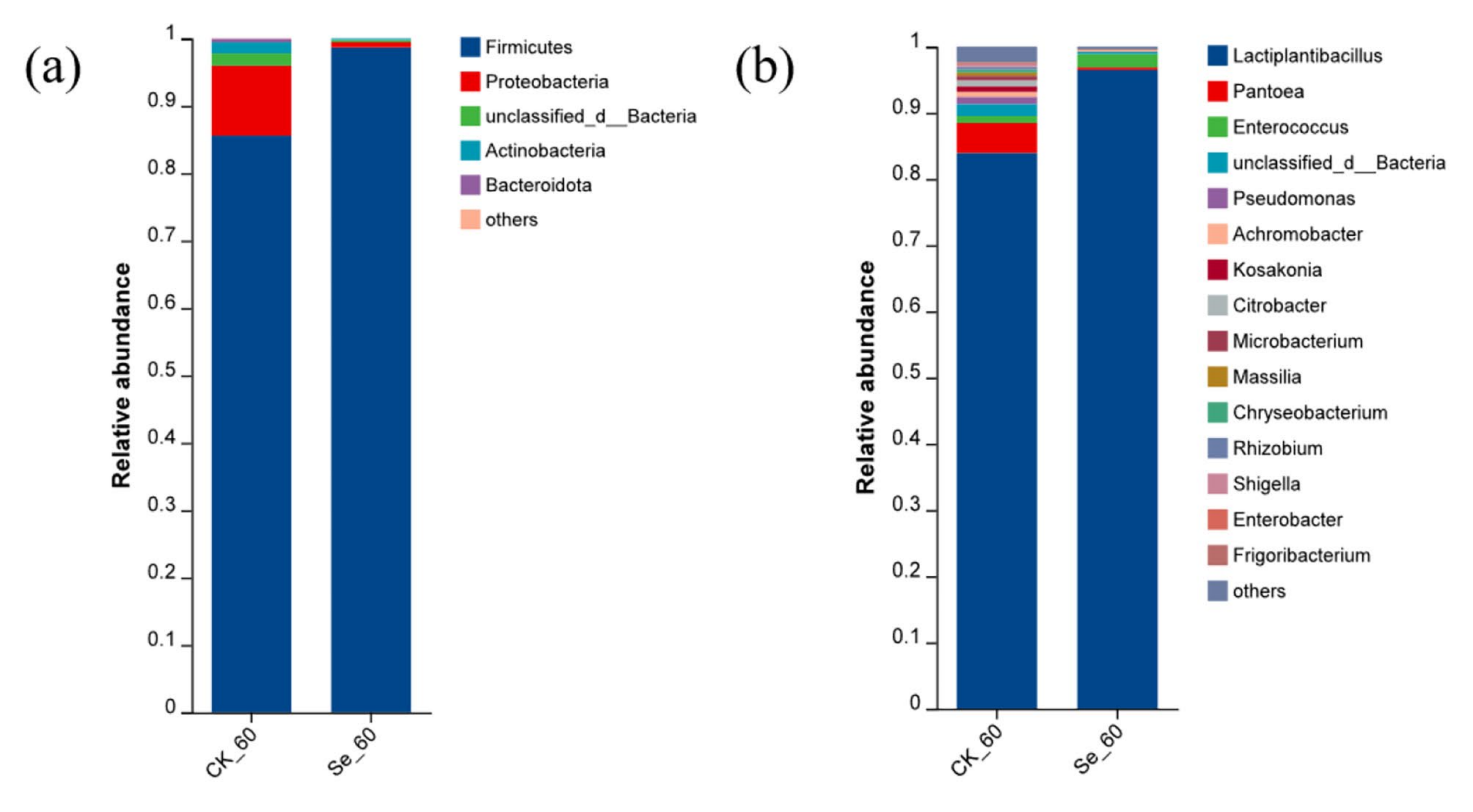
Bacterial community composition in alfalfa silage. (a), The bacterial community was shown at the phylum level; (b), The bacterial community was shown at the genus level. CK, not selenium-enriched; Se, selenium-enriched. 60, 60 days of ensiling

**Fig. 6:**
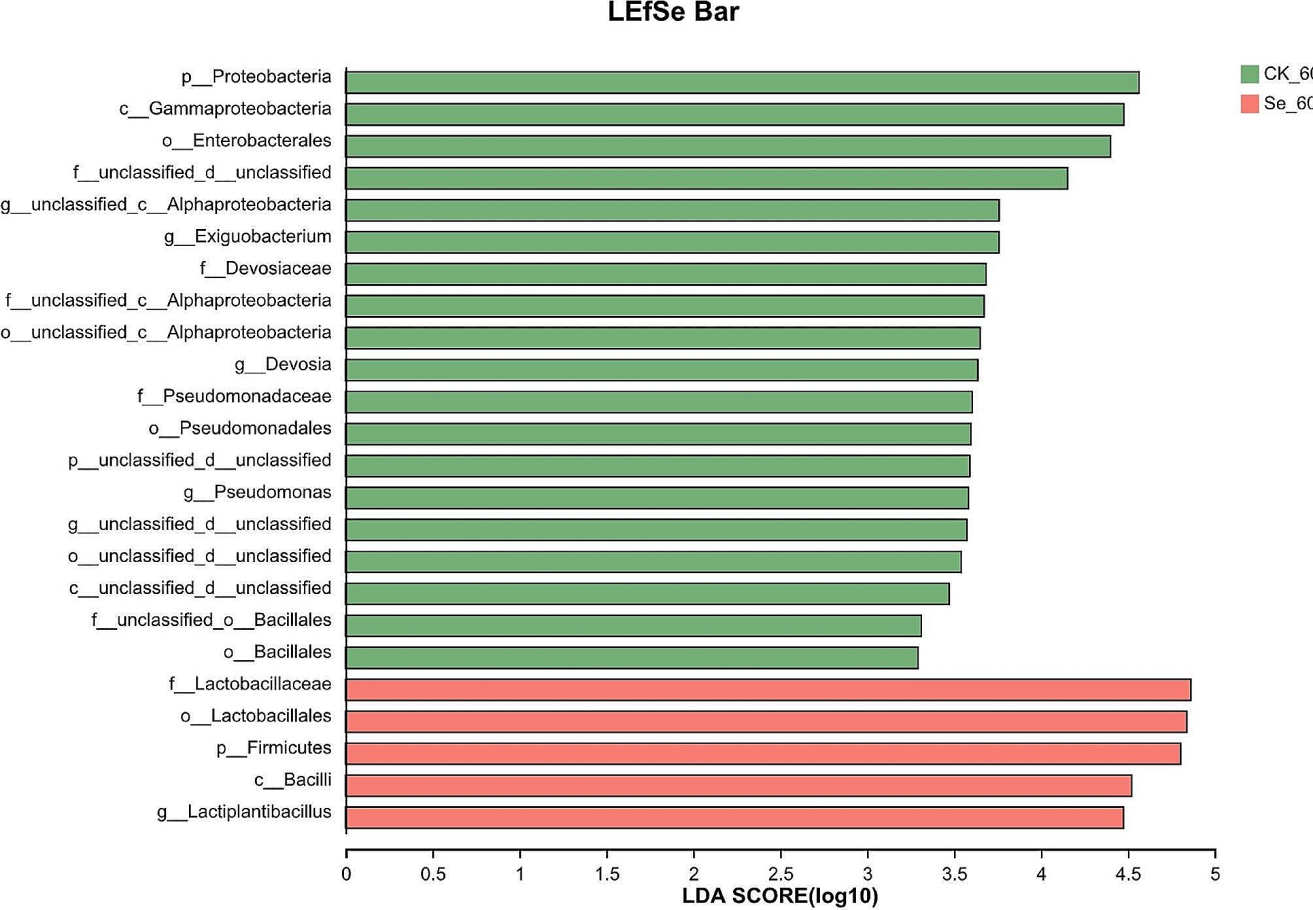
Comparison of microbial changes in selenium-enriched alfalfa silage using the LEfSe online tool. CK, not selenium-enriched; Se, selenium-enriched. 60, 60 days of ensiling

High-throughput sequencing methods were performed in variable regions 3 and 4 of 16s rDNA to calculate and assess bacterial diversity after silage (Fig. 4). Non-metric multidimensional scaling (NMDS) was used to analyze differences in microbial community distribution and structure between treatments. There were some differences in bacterial microbial diversity between the two treatment groups. The bacterial community composition between the two treatment groups for 60 days of silage is illustrated in Fig. 5. The microorganisms at the phylum level in the CK and Se treatment groups were mainly Firmicutes (85.58%, 98.68%) and Proteobacteria (10.38%, 0.79%). At the genus level, *Lactiplantibacillus* (83.95%, 96.49%), *Pantoea* (4.5%, 0.37%), *Enterococcus* (1.05%, 2.02%), *unclassified _ d _ Bacteria* (1.83%, 0.31%) and *Pseudomonas* (1.07%, 0.02%) were the main microorganisms in the CK and Se treatment groups, respectively. We used LEfSe to identify specific communities in the samples and only performed statistical analyses from the phylum to genus level in this study (Fig. 6). LEfSe verified LDA values of 2 or greater. The CK group was significantly enriched with 19 bacteria and Proteobacteria (4.56) had the largest LDA score, the Se group was significantly enriched with 5 bacteria and *Lactobacillaceae* (4.86) had the largest LDA score.

### In vitro ruminal fermentation

The in vitro digestibility of each chemical constituent of alfalfa silage for 60 d is shown in Table 4. IVNDMD, IVADFD, and IVNDFD were significantly higher in the Se group than in the CK group, whereas IVCPD had no significant effect. IVDMD, IVADFD and IVNDFD were 2.31%, 10.63% and 3.49% higher than CK in Se, respectively.

**Table 4:** Effect of selenium enrichment on in vitro digestion of alfalfa silage for 60 days

Items	CK	Se	SEM	*p*-value
IVCPD	86.88a	86.85a	0.21	0.951
IVDMD	61.12b	62.53a	0.35	0.012
IVADFD	30.58b	33.83a	0.74	0.001
IVNDFD	41.49b	42.94a	0.36	0.015

IVCPD: In vitro digestibility of proteins; (b), IVDMD: In vitro digestibility of dry matter; (b), IVADFD: In vitro digestibility of acid detergent fibers; (b), IVNDFD: In vitro digestibility of neutral detergent fibers. CK: not selenium-enriched; Se: selenium-enriched. Identical lowercase letters in the same row indicate no significant difference between selenium treatments at the 0.05 level (*p* > 0.05).

## Discussion

The characteristics of the silage material directly influence the fermentation characteristics of the silage [39]. Higher DM and WSC contents of silage raw materials are easy to be silage successfully, especially enough WSC content is an important factor for silage fermentation [40]. In this study, selenium enrichment measures significantly increased the DM, WSC and CP contents of alfalfa silage ingredients, which was similar to Xia et al [37]. The reason may be attributed to the application of exogenous selenium will, to a certain extent, promote the formation of selenium-containing small molecule proteins such as selenomethionine and other selenium-containing small molecule proteins in the plant, or promote the increase of other amino acids such as proline, arginine, etc., thus leading to the CP content of selenium-enriched alfalfa to be significantly higher than the CP content of CK [41, 42]. It is also reported that exogenous selenium will promote the photosynthesis of the plants and accelerate the accumulation of carbohydrates in the plants, leading to the increase of the DM content in alfalfa [43]. Fiber content has an important effect on livestock rumination and is used as an important indicator for evaluating forage quality. Lower cellulose content of forage means that it is of better quality and can be easily digested by livestock [44, 45]. In our study, we found that selenium enrichment measures significantly reduced the content of ADF and NDF in alfalfa silage material. The reason may be that the application of suitable exogenous selenium regulates the plant phenylpropane and flavonoid biosynthesis pathways, which may slow down the synthesis of lignin and other fibers in the plant itself, resulting in lower cellulose content [46, 47].

Good quality silage tends to have the lowest loss of nutrients during fermentation [48]. After 60 days of silage, the DM and WSC contents of the Se group were close to those of the CK, but the rate of loss was higher than that of the CK. The CP, ADF and NDF contents of the Se group were significantly higher than those of the CK group, however, the rate of loss of CP and ADF was higher than that of the CK group, and the rate of loss of NDF was slightly lower than that of the CK group. Moreover, for DM and CP in the Se group, the loss rate was greatest at the stage of 15 to 30 days of silage. The reason may be that the microbial activity in the Se group was inhibited by selenium as a whole during the first 15 days of silage, resulting in a slower silage fermentation process than that in the CK group, which may be alleviated with the gradual decrease of pH and slow metabolism of microorganisms [49]. After 15 days of silage lactic acid bacteria started to play their role to accelerate the whole fermentation process, which led to a greater loss of DM and CP by 30 days of silage. The biggest difference between NDF and ADF is that the former contains some hemicellulose. The different rates of ADF and NDF loss may be due to the effect of exogenous selenium on hemicellulose synthesis in alfalfa fresh samples. Alternatively, it may be due to the inhibition of the growth of some ferulic acid esterase-producing lactic acid bacteria during silage, leading to a decrease in the rate of NDF degradation [50, 51]. Although the rate of chemical loss during the silage process was greater in the Se group than in the CK group, in terms of the content of each chemical component retained at the completion of the final silage, it was better than that of the CK group as a whole, which shows that the alfalfa silage after selenium enrichment has a certain degree of feasibility. The rate and degree of pH decrease are considered to be the key signals reflecting the fermentation process of the silage [40, 52]. In this study, the pH of the Se group was significantly higher than that of the CK group at 15 days of silage and significantly lower than that of the CK group after 30 days of silage. This is due to the fact that the inoculated *Lactobacillus plantarum* mainly regulates the bacterial community in the early and middle stages of silage, and for the Se group, the inoculated *Lactobacillus plantarum* plays a later role than that of the CK group, which suggests the presence of selenium in the pre-silage period inhibits the fermentation of microorganisms [53, 54]. With the increase of silage time, lactic acid bacteria will gradually dominate, resulting in the production of a large amount of lactic acid, which leads to a rapid decrease in pH [55]. The change pattern of LA content in the present study confirms the above results to a certain extent. The change rule of NH_3_-N is inconsistent with the depletion of CP. From 15 to 30 days of silage, the rate of CP loss in the Se group was the largest, but the content of NH_3_-N was not significantly different from that of the CK group at 30 days of silage. It is possible that at this stage, some *Lactobacillus plantarum* may affect the content of NH_3_-N during protein decomposition or form some nitrogenous volatiles such as amines [56].

In recent years, many scholars have started to explore some biofunctional metabolites in silage, such as bacteriostatic, antioxidant and anti-inflammatory compounds and other functional components, which can improve the health and welfare of animals [57–59]. It is an interesting idea to start with silage ingredients and increase their own functional components to improve the nutritional value of silage and provide essential microcomponents for livestock. In the present study, exogenous selenium sprayed on alfalfa at the growth stage effectively increased the total, inorganic and organic selenium contents of fresh samples of silage alfalfa, with similar results to Kewen et al [60] and Di et al [61]. This is due to the fact that alfalfa itself belongs to a kind of legume with high selenium enrichment capacity, and as a natural converter, it can significantly absorb and transform exogenous selenium [13]. The appropriate dose of exogenous selenium can promote the growth and metabolism of the plant, which can stimulate the plant roots to absorb trace elements such as selenium, iron, magnesium and so on to a greater extent, thus promoting the absorption of selenium by the plant [37]. While selenium in alfalfa fresh samples in the Se group mainly existed in the form of SeMet, SeMeCys, the results of this study were consistent with the study of Dong et al [62]. In contrast, selenium in alfalfa fresh samples in the CK group mainly existed in the form of SeMeCys and SeCys_2_. The difference in the proportion of various organic selenium was due to the fact that the Se group was treated with foliar spraying of nano-selenium, while the CK group was in the natural state of normal absorption of inorganic selenium in the transformation of the soil, and both of them absorbed selenium sources of different types, different ways leading to different metabolic pathways [63]. We found that the various selenium contents in both treatment groups tended to decrease with increasing silage time, suggesting that some microorganisms degraded selenium in the silage. The degradation of selenium mainly occurred before 30 days of silage, in which some microorganisms might have decomposed selenium due to the intense activity of silage microorganisms, while after 30 days of silage, the whole microbial community of silage was in a stable state, and thus the degradation of selenium also gradually stabilized. It has been shown that some bacteria can biotransform selenium salts into volatile selenium compounds (diethylselenide, dimethylselenide and dimethyl diselenide) [64]. Therefore, the mechanism of how selenium is degraded needs to be further explored.

In this study, 16SrRNA sequencing was used to reveal the differential effects of selenium enrichment on bacterial diversity and community composition in alfalfa after silage. We found a difference in the bacterial diversity index between the Se and CK groups after 60 days of silage, with the former being lower than the latter. In terms of bacterial community abundance, the phylum Thick-walled Bacteria and the phylum Mycobacterium were predominant in alfalfa silage in this study. These findings are consistent with previous reports studying alfalfa silage [65]. Firmicutes and Proteobacteria were the most abundant phylum in fermented silage [12]. Mudasir et al. reported the transformation of bacterial community from Firmicutes to Proteobacteria and they revealed that anaerobic and acidic environments were able to sustain the growth of Firmicutes [66]. At 60 days of silage, Firmicutes were significantly higher in the Se group than in the CK group, while Proteobacteria were lower than in the CK group, as argued in our study. In terms of the genus level of bacteria, the highest abundance of *Lactobacillus* was found in both the Se and CK groups after 60 days of silage, and the former had an abundance of more than 98%, which was much higher than that of CK (85.58%). This also implies that after the addition of *Lactobacillus*, the Se group had a better fermentation effect than the CK group. *Lactobacillus* were the most dominant microorganisms in all silage samples at any point in time during the fermentation process, as shown in the study by Hao et al [67]. Increased lactic acid production, decreased pH and improved silage quality were all associated with greater abundance of lactic acid bacteria. This also explains why pH was significantly lower in the Se group than in the CK group during the 60-day silage period. The reason for such a high abundance of lactic acid bacteria could be that the overall loss of WSC was greater in the Se group than in the CK group, providing sufficient fermentation substrate for *Lactobacillus* fermentation. *Pantoea* was significantly higher in the CK group than in the Se group, and *Pantoea* is a parthenogenetic anaerobic bacterium that was present throughout the silage process [52]. This means that the presence of selenium somehow inhibits the colonization of *Pantoea* during silage fermentation. Overall, although the fermentation process in the Se group was slower than CK in the pre-silage stage, the final fermentation result of the Se group was more favorable.

The use of in vitro digestion for the determination of silage digestibility is widely used in the evaluation of forages due to its advantages such as low cost, rapidity and reproducibility, and the fact that it is performed in a controlled environment [68]. In this study, it was found that IVDMD, IVADFD, and IVNDFD were significantly higher in Se than in CK. The reason for this may be the change in the structure of ADF and NDF of selenium-enriched alfalfa after silage. Or the presence of selenium also improves the community structure of rumen microorganisms in livestock, leading to an increase in their digestibility. Whereas there was no significant effect on the digestibility of IVCPD, probably due to the fact that some of the selenium-containing proteins are released in the intestinal site and only a small part of them is broken down in the rumen [9]. This in turn ensures higher levels of rumen non-degradable proteins. It has been shown that increased levels of rumen non-degradable proteins increase the availability of nitrogen for microbial protein synthesis, thus increasing microbial activity and its ability to digest feed [69, 70]. Better rumen fermentation and microbial activity resulted in increased enzyme production, improved DM degradation, and reduced nutrient loss from the rumen [71]. This further explains the higher IVDMD in Se than CK. It has been found that increased microbial protein synthesis improves NH_3_ utilization and the effectiveness of fiber digestion, thus ensuring that the diet can be optimally utilized [72]. The increased level of rumen non-degradable proteins may also be responsible for the significantly higher IVADFD and IVNDFD in the Se group compared to CK.

## Conclusion

Nano-selenium can be absorbed by alfalfa and converted into organic selenium. During the process of silage fermentation, the presence of selenium can facilitate the growth of beneficial bacteria, such as *Lactobacillus*, and enhance the quality of alfalfa silage fermentation. Following a period of 60 days, the organic selenium present in alfalfa silage was predominantly found in the form of SeMet. Furthermore, selenium-enriched silage products demonstrated increased IVDMD, IVADFD and IVNDFD, which further substantiated the feasibility of selenium-enriched silage. The selenium content of the selenium-enriched product after silage (0.368 mg.kg^− 1^) was found to be within the safe dose range for livestock (0.30-1.00 mg.kg^− 1^). This study provides a foundation for the utilisation of selenium-enriched silage products. Nevertheless, the specific mechanism of action of selenium in silage requires further investigation.

## Data Availability

The datasets generated or analysed during the current study are available from the corresponding author upon reasonable request. Sequencing data for 16 S rRNA gene sequence were stored in NCBI (https://www.ncbi.nlm.nih.gov/) with BioProject accession number PRJNA1099316.
