# Correction: Effects of selenium enrichment on fermentation characteristics, selenium content and microbial community of alfalfa silage

**DOI:** 10.1186/s12870-024-05308-w

**Published:** 2024-07-03

**Authors:** Pengbo Sun, Gentu Ge, Lin Sun, Shuai Du, Yichao Liu, Xingquan Yan, Jiawei Zhang, Yuhan Zhang, Zhijun Wang, Yushan Jia

**Affiliations:** 1 Key Laboratory of Forage Cultivation, Processing and High Efficient Utilization, Ministry of Agriculture, Beijing, People’s Republic of China; 2 Key Laboratory of Grassland Resources, Ministry of Education, Beijing, People’s Republic of China; 3 College of Grassland, Resources and Environment, Inner Mongolia Agricultural University, Hohhot, China; 4 Inner Mongolia Academy of Agricultural and Animal Husbandry Sciences, Hohhot, China; 5 Ordos Institute of Forestry and Grassland Science, Ordos, China; 6 Forestry and Grassland Work Station of Inner Mongolia, Hohhot, China

**Correction: BMC Plant Biol 24, 555 (2024)**

10.1186/s12870-024-05268-1

Following publication of the original article [1], the authors identified an error Fig. 4. The panels of Fig. 4b and Fig. 4c are the same. The correct figure is given below:

Incorrect Figure 4.

**Fig. 4:**
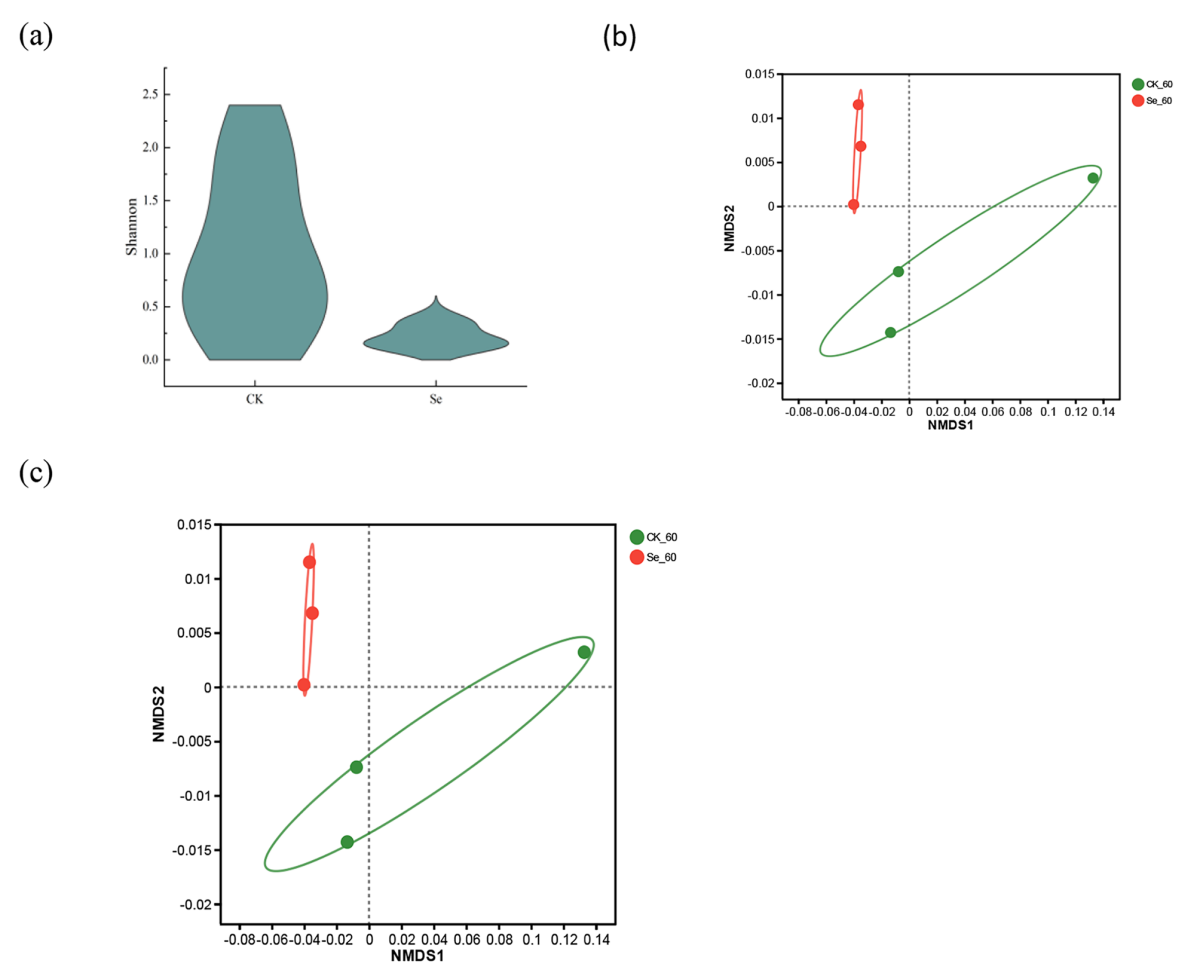
Analysis of bacterial diversity in alfalfa silage. (**a**), Shannon of bacterial alpha diversity; (**b**), Analysis of bacterial NMDS in alfalfa silage; (**c**), Bacterial OUT Venn in alfalfa silage. CK, not selenium-enriched; Se, selenium-enriched. 60, 60 days of ensiling

Correct Figure 4.

**Fig. 4:**
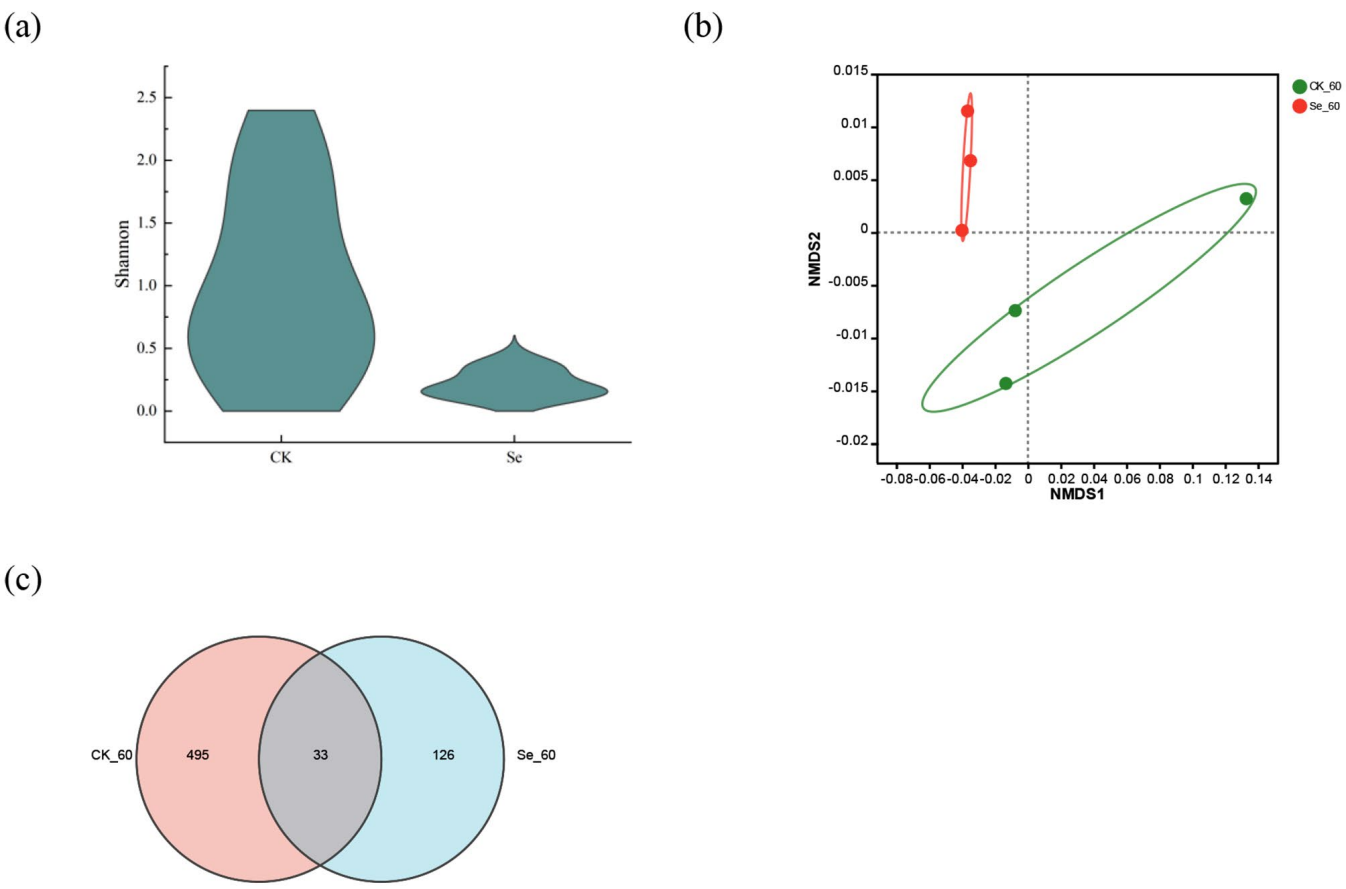
Analysis of bacterial diversity in alfalfa silage. (**a**), Shannon of bacterial alpha diversity; (**b**), Analysis of bacterial NMDS in alfalfa silage; (**c**), Bacterial OUT Venn in alfalfa silage. CK, not selenium-enriched; Se, selenium-enriched. 60, 60 days of ensiling

The original article [1] has been corrected.
